# Why does such a cyst appear after *unilateral biportal* endoscopy surgery: A case report and literature review

**DOI:** 10.1097/MD.0000000000036665

**Published:** 2023-12-15

**Authors:** Xiulong Lou, Penglei Chen, Jing Shen, Jie Chen, Yuying Ge, WeiFeng Ji

**Affiliations:** a Zhejiang Chinese Medical University, Hangzhou, China; b The First Affiliated Hospital of Zhejiang Chinese Medical University, Hangzhou, China.

**Keywords:** arachnoid cyst, case report, lumbar disc herniation, unilateral biportal endoscopy (UBE)

## Abstract

**Background:**

*Unilateral biportal endoscopy* (UBE) has been widely and skillfully used in the treatment of lumbar disc herniation and spinal canal stenosis. UBE surgery also brings some complications, such as dural tear, epidural hematoma, residual nucleus pulposus, etc. And we found a rare case of arachnoid cyst after UBE.

**Case presentation:**

A 48 years old female who had a history of cholecystectomy, nephrolithiasis, hyperthyroidism, chronic atrophic gastritis, and colonic polyps with several years of low back pain and numbness in both lower limbs was found have arachnoid cyst 3 years after UBE operation. We hope that we can give a new aspect of complication after the UBE treatment in the future.

**Conclusion:**

We believe that the postoperative hypertension and the lack of postoperative back muscle strength training and some personal factors are the possible reasons for the arachnoid cyst in this case.

## 1. Introduction

Due to the trend of population aging and the change of modern lifestyle, there are more and more patients with lumbar disc herniation, and they are becoming younger and younger. The treatment of lumbar disc herniation mainly includes conservative treatment and surgical treatment. Conservative treatment includes physical therapy, exercise therapy, behavior therapy, orthosis, band, acupuncture, manipulation and drug logic intervention. Surgical treatment included discectomy, laminectomy, and interbody fusion. The advantages of UBE include less bleeding, better visualization, and the ability to be used in decompression and fusion procedures. Studies have shown that the incidence of postoperative complications after UBE is about 6.7%.^[1]^ Although UBE, as a minimally invasive surgery, brings fewer postoperative complications than traditional open surgery, there are still some complications, such as dural injury and nerve injury, which may affect the future life of patients.

## 2. Case presentations

**Figure 1.**
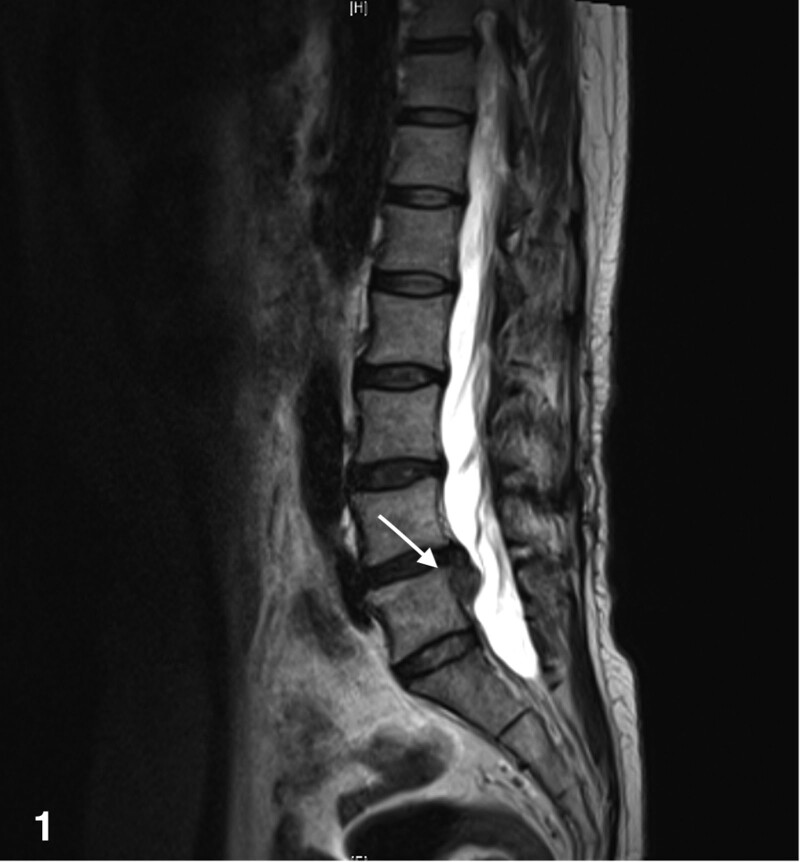
Sagittal MRI of lumbar spine in patients before UBE surgery. MRI = magnetic resonance imaging.

**Figure 2.**
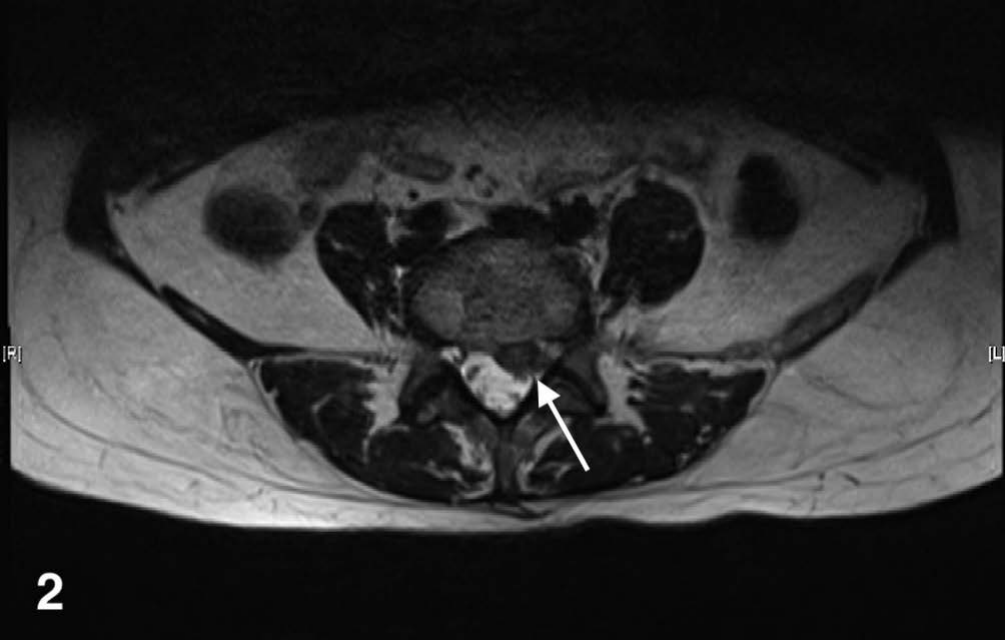
MRI horizontal plane of lumbar spine before UBE surgery. MRI = magnetic resonance imaging.

The patient is a 48-year-old Asian woman with low back pain and numbness in both lower limbs for 2 months and has history of cholecystectomy, nephrolithiasis, hyperthyroidism, chronic atrophic gastritis, and colonic polyps. Lumbar magnetic resonance imaging detected left posterior protrusion of the lumbar 5-sacral 1 disc with left inferolateral detachment of the nucleus pulposus, and local spinal canal secondary stenosis (Figs. 1 and 2). At the outset the patient was treated due to paroxysmal palpitation. The orthopedics doctor performed percutaneous Lumbar 4/5 transforaminal endoscopic discectomy + laminoplasty + radiofrequency ablation under general anesthesia. The procedure went smoothly. The patient blood pressure fluctuates dramatically and then she was admitted to intensive care unit after surgery.

After the patient was transferred to the general ward, the relevant examinations and treatments were repeated, when the condition was stable, the patient was discharged with medicine. The pathological examination of the lumbar spine reported that the Lumbar 4/5 intervertebral disc had degenerated fibrocartilage tissue. During the whole postoperative treatment, functional rehabilitation exercises of the back muscles were not performed.

**Figure 3.**
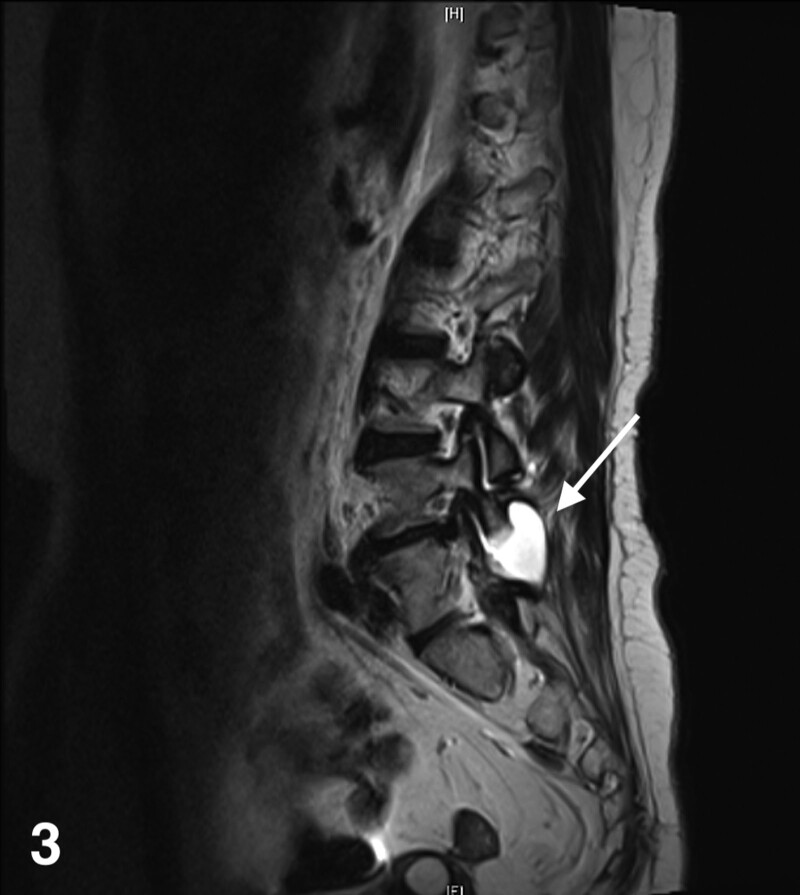
Sagittal MRI of lumbar spine in patients after UBE surgery. MRI = magnetic resonance imaging.

**Figure 4.**
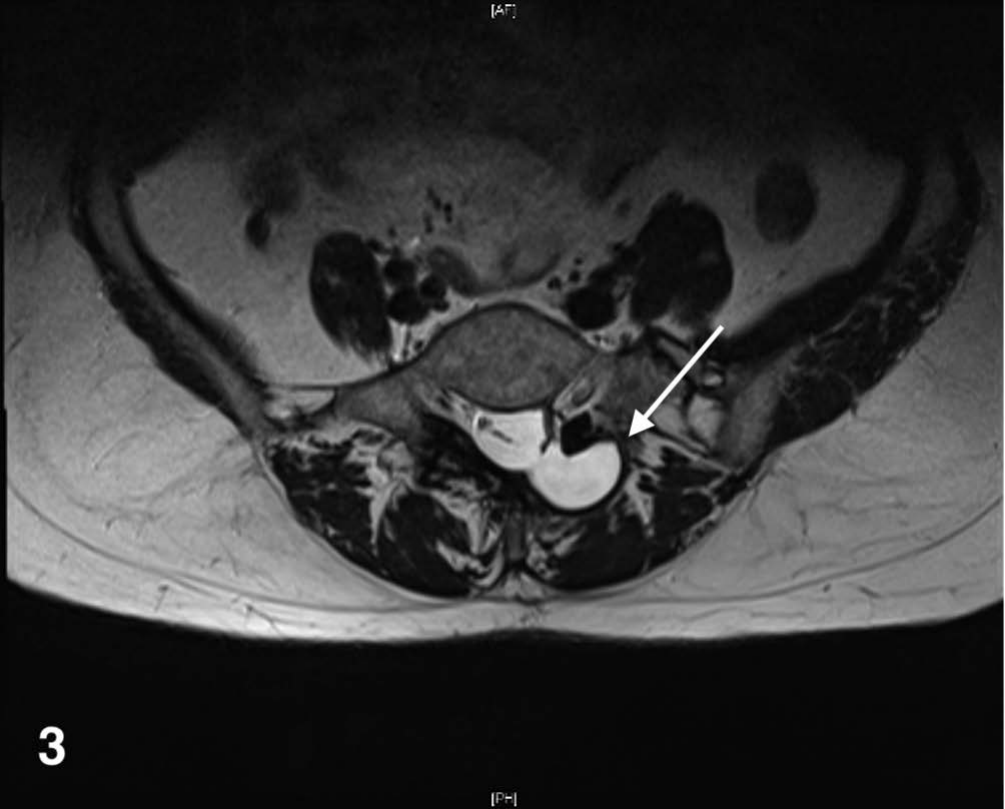
MRI horizontal plane of lumbar spine after UBE surgery. MRI = magnetic resonance imaging.

In January 2023, the patient was admitted for treatment of “unstable blood pressure control.” During the treatment, the patient developed pain after sitting down after UBE 3 years ago, and then underwent lumbar magnetic resonance imaging examination again. The examination results showed: Small patches of abnormal signals in the local soft tissue on the dorsal side of L4 vertebral body, considering the possibility of cysts, please combine with clinical practice (Figs. 3 and 4).

## 3. Discussion

### 3.1. The research development of UBE

The UBE procedure was first proposed and reported by De Antoni in 1996. However, UBE was suppressed by the development of transforaminal endoscopy, and became popular again after Soliman treated 43 patients with lumbar disc herniation with UBE in 2013. Studies have shown that South Korea has provided a huge support force in the development of UBE, represented by Chio and others.^[2]^ Zuo et al hold that hidden blood loss of UBE surgical patients should be not ignored.^[3]^ Although UBE may inferior to other spinal endoscopic techniques, it has its own merits which can make it stand out in comparison to the conventional surgeries, for example, the smaller surgical incision, the less pain of patients early after operation, the flexible operation, its wide vision and short learning curve which are worth promoting in primary hospitals.^[4]^

### 3.2. Research on complications of UBE

There are a number of complications after UBE, Examples include dural tear, epidural hematoma, retroperitoneal fluid collection, nucleus pulposus residue, nerve root injury and infection. Zhang et al hold that as a typical representative of minimally invasive techniques, UBE still needs to complete extensive decompression and tissue resection in a narrow working space, these are resulting in many surgery-related injuries. Therefore, familiarity with the anatomy of the lumbar spine is essential to reduce complication.^[5]^ Dural injury is the most common complication after UBE surgery. Due to the narrowing of the space between the dural sac and the lamina, the adhesion between the dural sac and ligamentum flavum (LF) forms a blind area, and the ligament structure between the dural sac and the surrounding vertebral canal wall, the meningovertebral ligament and LF. Thus, pulling on the LF can avulse the LF along with part of the dorsal dural sac and small vessels, resulting in dural tears and, when the lamina is removed using a Callison punch, may tear along with the dural sac.^[5,6,7]^ Epidural hematoma is also a common complication after UBE. The symptoms of epidural hematoma usually appear within 24 hours after the operation, but about 43% of cases do not appear until 4 days or later after the operation.^[8]^ Fujiwara et al^[9]^ hypertension is reported to be a cause of epidural hematoma after spinal surgery. Studies have also reported that large gelatin sponges placed on the dura during surgery and the discharge of postoperative drainage fluid are also causes of postoperative epidural hematoma.^[9]^ In addition, we found that multilevel surgery, bleeding from branches of the internal vertebral venous plexus, excessive blood loss, preoperative coagulopathy, advanced age, use of NSAIDs, Rh-positive blood type, low hemoglobin level, and alcohol consumption were associated with the occurrence of this symptom.^[7]^ In addition, improper operation can also lead to complications such as residual nucleus pulposus and nerve root injury.^[10,11]^

### 3.3. Arachnoid cyst and lumbar operation

We have never found the case report about arachnoid cyst after UBE surgery in the already rare reports. This is the reason that we intend to report this agnogenic case. An arachnoid cyst is a cystic cavity formed by the arachnoid membrane and contains cerebrospinal fluid-like fluid, which is a benign lesion. It is divided into 2 types: primary and secondary clinically. According to the location of arachnoid cysts, they are divided into intracranial arachnoid cysts and spinal cord arachnoid cysts. However, we have found that other traditional lumbar spine surgery can cause this symptom, although there are few reports of such a symptom. In 2013, Weng et al^[12]^ reported a 56-year-old patient who developed numbness in the right lower limb 3 years after L4/L5 nerve root decompression, pedicle screw internal fixation, and posterolateral bone graft fusion. The report did not describe the patient in detail, only the cause of the arachnoid cyst. In 2016, Nath et al^[13]^ reported a case of intradural arachnoid cyst after laminectomy. He suggested that laminectomy, which can usually be the method of removing an arachnoid cyst, could be the cause of a secondary intradural extramedullary arachnoid cyst. In 2019, Yokoyama et al^[14]^ reported a case of subdural subarachnoid hematoma and cauda equina nerve injury after microsurgical decompression of the lumbar spine. They believed that the potential adhesion of the arachnoid membrane and the decompression effect of the operation may create pores in the arachnoid membrane and the check valve, which were the 2 reasons for the postoperative arachnoid cyst of the patient. Yuen et al^[15]^ first studied the incidence of intradural arachnoid cysts in lumbar spine patients after surgery, and they believed that the phenomenon of intradural arachnoid cysts after surgery was more common in our expectation, and it was a reason for the persistence of postoperative pain. In 2022, Kawasaki et al^[16]^ reported a case of asymptomatic subdural arachnoid cyst after laminectomy for lumbar spinal stenosis, and they suggested that it is very rare to develop a postoperative subdural arachnoid cyst without a dural incision during surgery. This is also in line with the study by Yuen et al.

There are no reports of arachnoid cysts after UBE, but the related complications of spinal surgery under traditional surgery are reference. Improper operation in the process of UBE can also lead to the redistribution of cerebrospinal fluid pressure and dural injury, which will lead to the appearance of postoperative arachnoid cysts.

### 3.4. To explore the cause of arachnoid cyst in this case

First, we considered that the patient blood pressure was consistently unstable and that hypertension was one of the causes of epidural cysts.^[17]^ We reviewed relevant data, arachnoid cysts are cysts formed by arachnoid membranes, containing cerebrospinal fluid-like fluid. Studies have shown that intracranial pressure and cerebrospinal fluid pressure at the lumbar spine are increased in patients with hypertension.^[18,19]^ Therefore, we believe that hypertension is the primary potential factor for the occurrence of arachnoid cyst after surgery in this patient.

Secondly, this patient is a little different from other patients after UBE. Other patients are admitted to the orthopedic ward after the operation, and our orthopedic doctors will advise them to exercise the strength of the back muscles after the operation to speed up the recovery. However, the patient in this case did not perform postoperative lumbar dorsal muscle exercise. Therefore, we believe that the postoperative cyst is related to postoperative exercise and rehabilitation. Pourahmadi et al^[20]^ believe that motor control training has a beneficial effect on long-term postoperative outcomes compared with conventional rehabilitation. At a follow-up visit 3 years later, the patient reported pain in the lower limbs when sitting down after surgery, which was most likely related to a lack of adequate postoperative exercise.

Finally, during follow-up, we learned that the patient had been taking orlistat before her UBE procedure in 2020, but she had not informed the surgeon of this. However, Sahebkar et al^[21]^ concluded that orlistat had a significant reduction in systolic and diastolic blood pressure in its users. This is contrary to the hypothesis that hypertension causes alterations in CSF dynamics. We cannot rule out the possibility that the patient had a physical cause, or that trauma was received during the first 3 years after surgery, or other unknown causes may have caused the present arachnoid cyst. Readers can also express their own views to improve our inadequate medical knowledge. We hope that this case report will attract the attention of experts in related fields to increase the knowledge content of UBE surgery, give a better quality of life to lumbar disc patients and other related patients, and optimize the relationship between doctors and patients.

## Acknowledgments

This article is distributed under the terms of the Creative Commons Attribution License which permits any use, distribution and reproduction in any medium, provided the original author(s) and source are credited.

## Author contributions

**Supervision:** WeiFeng Ji.

**Writing – original draft:** Xiulong Lou.

**Writing – review & editing:** Penglei Chen, Jing Shen, Jie Chen, Yuying Ge, WeiFeng Ji.

## References

[1] Lin GX Huang P Kotheeranurak V. A systematic review of unilateral biportal endoscopic spinal surgery: preliminary clinical results and complications. World Neurosurg. 2019;125:425–32. 30797907 10.1016/j.wneu.2019.02.038

[2] Chu P Wang T Zheng J. Global and current research trends of unilateral biportal endoscopy/biportal endoscopic spinal surgery in the treatment of lumbar degenerative diseases: a bibliometric and visualization study. Orthop Surg. 2022;14:635–43. 35293686 10.1111/os.13216 PMC9002063

[3] Zuo R Jiang Y Ma M. The clinical efficacy of biportal endoscopy is comparable to that of uniportal endoscopy via the interlaminar approach for the treatment of L5/S1 lumbar disc herniation. Front Surg. 2022;9:1014033. 36238864 10.3389/fsurg.2022.1014033 PMC9553067

[4] Guo S Tan H Meng H. Risk factors for hidden blood loss in unilateral biportal endoscopic lumbar spine surgery. Front Surg. 2022;9:966197. 36046261 10.3389/fsurg.2022.966197 PMC9420975

[5] Zhang Q Wei Y Wen L. An overview of lumbar anatomy with an emphasis on unilateral biportal endoscopic techniques: a review. Medicine (Baltimore). 2022;101:e31809. 36482646 10.1097/MD.0000000000031809 PMC9726330

[6] Uchikado H Nishimura Y Hattori G. Micro-anatomical structures of the lumbar intervertebral foramen for full-endoscopic spine surgery: review of the literatures. J Spine Surg. 2020;6:405–14. 32656378 10.21037/jss.2019.10.07 PMC7340827

[7] Kim JE Choi DJ Park EJ. Risk factors and options of management for an incidental dural tear in biportal endoscopic spine surgery. Asian Spine J. 2020;14:790–800. 32429015 10.31616/asj.2019.0297 PMC7788375

[8] Anno M Yamazaki T Hara N. The incidence, clinical features, and a comparison between early and delayed onset of postoperative spinal epidural hematoma. Spine. 2019;44:420–3. 30095797 10.1097/BRS.0000000000002838

[9] Fujiwara Y Manabe H Izumi B. The impact of hypertension on the occurrence of postoperative spinal epidural hematoma following single level microscopic posterior lumbar decompression surgery in a single institute. Eur Spine J. 2017;26:2606–15. 28597302 10.1007/s00586-017-5165-9

[10] Kao FC Tsai TT Chen LH. Symptomatic epidural hematoma after lumbar decompression surgery. Eur Spine J. 2015;24:348–57. 24760464 10.1007/s00586-014-3297-8

[11] Zhang Y Tian L Hu P. [Research progress of unilateral biportal endoscopy technique in treatment of lumbar related diseases]. Zhongguo xiu fu chong jian wai ke za zhi. 2022;36:1234–40. 36310460 10.7507/1002-1892.202205087 PMC9626282

[12] Weng RHK Chang MC Feng SW. Progressive growth of arachnoid cysts with cauda equina syndrome after lumbar spine surgery. J Chin Med Assoc. 2013;76:527–31. 23806807 10.1016/j.jcma.2013.05.011

[13] Nath PC Mishra SS Deo RC. Intradural spinal arachnoid cyst: a long-term postlaminectomy complication: a case report and review of the literature. World Neurosurg. 2016;85:367.e1–4. 10.1016/j.wneu.2015.09.058 26428320

[14] Yokoyama K Yamada M Tanaka H. A case of dural herniation of the cauda equina caused by enlarged spinal subdural extra-arachnoid hygroma following lumbar microsurgical decompression: case report. NMC Case Rep J. 2021;8:261–5. 35079473 10.2176/nmccrj.cr.2020-0301 PMC8769481

[15] Yuen J Mcgavin L Adams W. Intradural symptomatic arachnoid cyst formation following non-instrumented lumbar decompression. Br J Neurosurg. 2021;35:352–7. 32924618 10.1080/02688697.2020.1817313

[16] Kawasaki T Takayama M Maki Y. Asymptomatic spinal subdural epiarachnoid hygroma after lumbar laminectomy for lumbar spinal canal stenosis: illustrative case. J Neurosurg Case Lessons. 2022;3:CASE2285. 36303483 10.3171/CASE2285 PMC9379644

[17] Heo DH Sharma S Park CK. Endoscopic treatment of extraforaminal entrapment of L5 nerve root (Far Out Syndrome) by unilateral biportal endoscopic approach: technical report and preliminary clinical results. Neurospine. 2019;16:130–7. 30943715 10.14245/ns.1938026.013 PMC6449829

[18] Ichinose T Miyashita K Tanaka S. Recurrent spinal intramedullary arachnoid cyst: case report and literature review. World Neurosurg. 2020;138:68–72. 32142944 10.1016/j.wneu.2020.02.106

[19] Yilmaz TF Aralasmak A Toprak H. Evaluation of CSF flow metrics in patients with communicating hydrocephalus and idiopathic intracranial hypertension. Radiol Med. 2019;124:382–91. 30560499 10.1007/s11547-018-0979-z

[20] Pourahmadi M Delavari S Hayden JA. Does motor control training improve pain and function in adults with symptomatic lumbar disc herniation? A systematic review and meta-analysis of 861 subjects in 16 trials. Br J Sports Med. 2022:bjsports-2021-104926. 10.1136/bjsports-2021-104926 35701082

[21] Sahebkar A Simental-Mendía LE Kovanen PT. Effects of orlistat on blood pressure: a systematic review and meta-analysis of 27 randomized controlled clinical trials. J Am Soc Hypertens. 2018;12:80–96. 29275922 10.1016/j.jash.2017.12.002

